# Correction to “Blood glucose screening in dental clinics as an opportunity for detection of diabetes and prediabetes: The Kyoutou Dental and Diabetes (KDD) Study”

**DOI:** 10.1111/jdi.70219

**Published:** 2025-12-09

Harai N, Tawata M, Nakamura H, *et al*. Blood glucose screening in dental clinics as an opportunity for detection of diabetes and prediabetes: The Kyoutou Dental and Diabetes (KDD) Study. *J Diabetes Investig*. 2025;16(9):1742–1749. https://doi.org/10.1111/jdi.70093

The author identified an unintentional labelling error in Figure 3 after the first online publication. The table calculates the total score, and the graph illustrates the sensitivity and specificity of each total score. The value for ‘alveolar bone loss’ was incorrectly captured as 14%; it has now been corrected to 11%. This correction does not affect the analyses, results, or conclusions.

**Figure 3:**
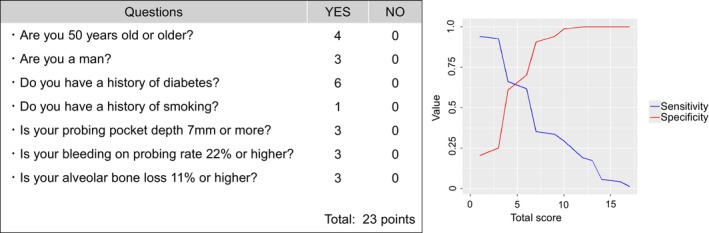
Scoring sheet for evaluating blood glucose screening positivity. This table calculates the total score, and the graph illustrates the sensitivity and specificity of each total score.

We apologize for the error.

